# Review of sheep breeding and genetic research in Türkiye

**DOI:** 10.3389/fgene.2024.1308113

**Published:** 2024-01-25

**Authors:** Kenan Burak Aydin, Ye Bi, Luiz F. Brito, Zafer Ulutaş, Gota Morota

**Affiliations:** ^1^ School of Animal Sciences, Virginia Tech, Blacksburg, VA, United States; ^2^ Department of Animal Sciences, Purdue University, West Lafayette, IN, United States; ^3^ Department of Animal Science, Faculty of Agriculture, Ondokuz Mayis University, Samsun, Türkiye; ^4^ Center for Advanced Innovation in Agriculture, Virginia Tech, Blacksburg, VA, United States

**Keywords:** association analysis, breeding, genomic selection, small ruminant, sheep

## Abstract

The livestock industry in Türkiye is vital to the country’s agricultural sector and economy. In particular, sheep products are an important source of income and livelihood for many Turkish smallholder farmers in semi-arid and highland areas. Türkiye is one of the largest sheep producers in the world and its sheep production system is heavily dependent on indigenous breeds. Given the importance of the sheep industry in Türkiye, a systematic literature review on sheep breeding and genetic improvement in the country is needed for the development and optimization of sheep breeding programs using modern approaches, such as genomic selection. Therefore, we conducted a comprehensive literature review on the current characteristics of sheep populations and farms based on the most up-to-date census data and breeding and genetic studies obtained from scientific articles. The number of sheep has increased in recent years, mainly due to the state’s policy of supporting livestock farming and the increase in consumer demand for sheep dairy products with high nutritional and health benefits. Most of the genetic studies on indigenous Turkish sheep have been limited to specific traits and breeds. The use of genomics was found to be incipient, with genomic analysis applied to only two major breeds for heritability or genome-wide association studies. The scope of heritability and genome-wide association studies should be expanded to include traits and breeds that have received little or no attention. It is also worth revisiting genetic diversity studies using genome-wide single nucleotide polymorphism markers. Although there was no report of genomic selection in Turkish sheep to date, genomics could contribute to overcoming the difficulties of implementing traditional pedigree-based breeding programs that require accurate pedigree recording. As indigenous sheep breeds are better adapted to the local environmental conditions, the proper use of breeding strategies will contribute to increased income, food security, and reduced environmental footprint in a sustainable manner.

## Introduction

Livestock farming is vital to Turkey’s agricultural sector and economy. Türkiye (Turkey) has extensive areas of meadows, pastures, forests, woodland, and arable land, which give it a great capacity to support a variety of livestock and crops (Akbay and Boz, 2005). Livestock production contributes about 25% of the total value of agricultural production and helps in the economic growth of rural households (Akbay and Boz, 2005). Small ruminant products provide an important source of income and livelihood for numerous farmers and households living in resource-poor areas in highlands and semi-arid territories.

Sheep (*Ovis aries*) are multipurpose animals that produce meat, milk, and wool (Zygoyiannis, 2006). Sheep are thought to have been domesticated in the Fertile Crescent about 10,000 years ago (Zeder, 2008). Sheep are the second known animal to have been domesticated, following the dog. Sheep production is one of the most important agricultural sectors in Türkiye due to the Turkish dietary preference (Ankarali, 1988). Türkiye is one of the largest sheep producers in Europe and West Asia and has a sheep production system that heavily relies on indigenous breeds. However, in recent years, the number of European breeds imported for dairy and meat production has increased. These sheep breeds are raised in almost all regions of Türkiye, including harsh environmental areas. The Turkish sheep breeds are well adapted to rangelands and can endure droughts and unstable nutrient availability (Ankarali, 1988). For this reason, sheep have been meeting human needs for many years by providing meat, milk, and fleece. They are able to convert grasses on poor rangelands into valuable sources of protein and wool. Sheep meat and milk account for 25% and 5% of domestic production, respectively (Turkish Statistical Institute, 2022). Sheep are commonly reared in extensive systems where ewes give birth once a year and graze on low-input and low-output grasslands unsuitable for cultivation. Due to the high feed costs, grazing plays an important role in Turkish farming. Sheep breeders prefer to raise sheep because they benefit from the forage in the grass and other species, such as cattle, cannot adapt well to harsh environmental conditions. In Türkiye, most sheep are raised for meat production, and the average consumption of 4.2 kg per person makes it an important source of red meat production, especially under harsh climate conditions (Şirin et al., 2017). Lambs are typically slaughtered when they reach 50–55 kg, resulting in a carcass weight of 18–20 kg. Most sheep milk is used in cheese production, with an average cheese consumption of 8.8 kg per person per year. Ewes are typically milked manually for about 3–4 months after weaning (Gürsoy, 2006). Despite the crucial role that sheep production plays in Turkey’s agricultural production and economy, a comprehensive review focused on sheep breeding and genetics in Türkiye has not been documented yet. A comprehensive literature review could guide the development of more effective breeding strategies incorporating modern methods and technologies.

Therefore, the primary objective of this paper was to comprehensively review the literature on sheep breeding and genetics in Türkiye for the past 30 years. This review is based on a thorough literature search using recent government statistics, Web of Science, and Google Scholar databases. The structure of this review article is as follows. First, we examine the current size of the indigenous sheep populations, the geographic locations where they are being raised, and their management practices based on the most recent data available. Next, we review some of the primary sheep breeds in Türkiye, their key characteristics, and their level of population genetic diversity. We also compiled the estimates of genetic parameters and genome-wide association study (GWAS) analyses of economically important traits. Lastly, an overall discussion and suggestions of next steps to be taken for improving sheep breeding and genetics in Türkiye are presented in the last section as concluding remarks.

## Sheep population demographics

**FIGURE 1:**
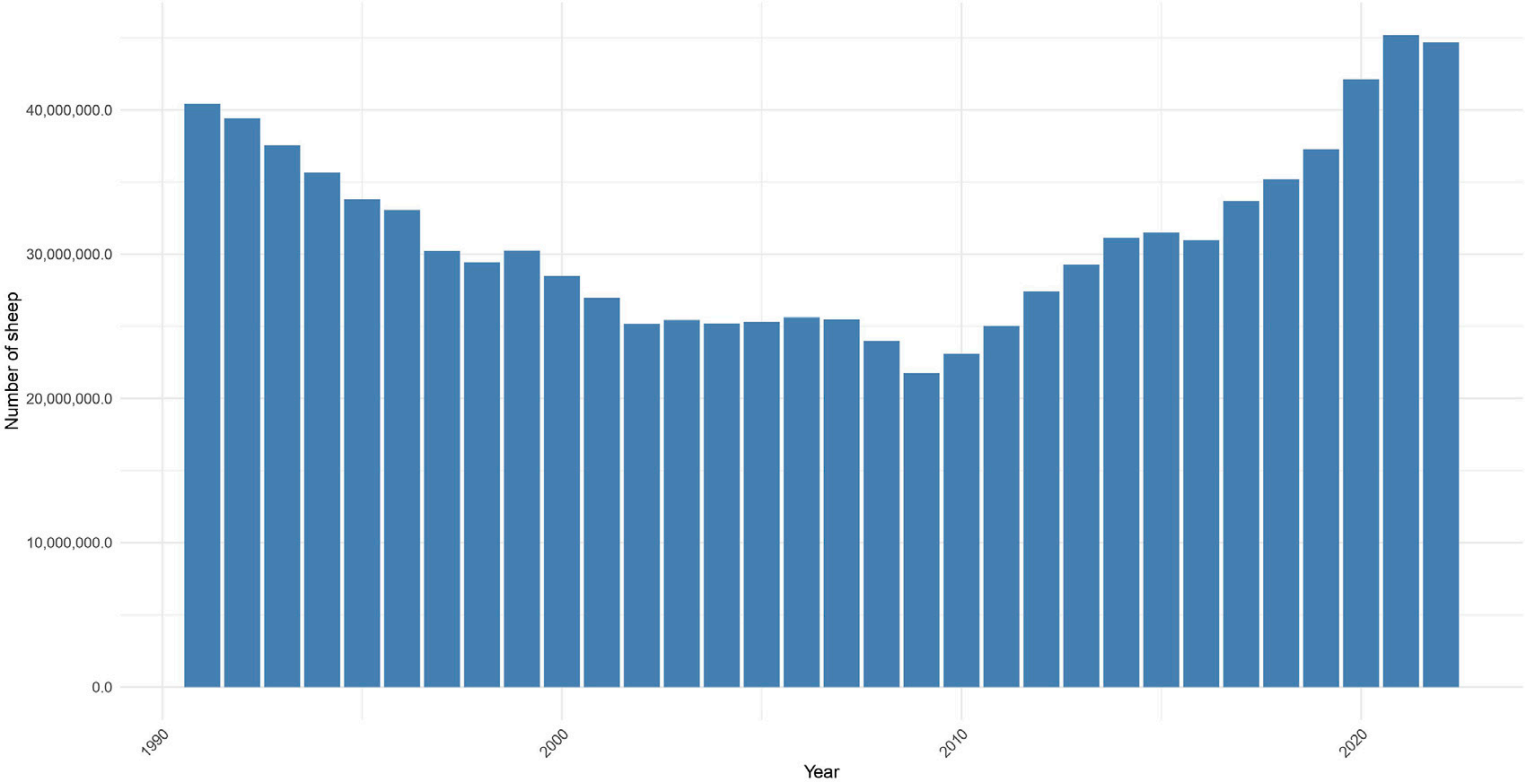
Bar graph showing the total number of sheep in the last 30 years in Türkiye. The 2022 data were obtained from the Turkish Statistical Institute.

Figure 1 shows the total number of sheep during the last 30 years in Türkiye (Turkish Statistical Institute, 2022). At present, there are 45 million sheep in the country, where approximately 83% of them are ewes. While the sheep population experienced a steady decline from 1990 to 2002 and remained fairly constant for a few years thereafter, it sharply rose to 45 million later. Several factors have contributed to the decline of the sheep population between 1990 and 2002. These include 1) decrease in pasture and rangeland areas due to conversion to crop production, 2) lower productivity of indigenous sheep breeds, 3) migration of population from rural to urban areas, 4) reluctance of farmers to adopt new technologies, 5) greater government support for poultry and dairy production as opposed to sheep production, and 6) lack of government support policies for sheep production (Gürsoy, 2006). In particular, sheep were replaced by dairy cattle in resource-rich areas and sheep production was forced to move to more remote, resource-limited, and arid areas (Ocak et al., 2010). The combination of these factors has led to the decline of the sheep industry in Türkiye.

The more recent increase in the sheep population after 2010 is due to the implementation of government support policies. To enhance the profitability of livestock production and improve livelihoods, the government introduced support policies in 2000 and 2005. These policies promoted meat and milk production. For example, livestock support increased from 19 million in 2000 to 1.16 billion dollars in 2015 (Erturk et al., 2015). Likewise, the share of livestock support in total agricultural support was 1.29% in 2000, but increased to 21.8% in 2015. These support policies have strengthened the sheep industry by providing support and incentives to livestock farmers. These policies have contributed to the growth and development of the sector, leading to an increase in the sheep population in Türkiye (Erturk et al., 2015). Another factor contributing to this trend is the recent rise in consumer demand for dairy products that provide significant nutritional and health benefits, such as cheese, yogurt, ice cream, butter, ayran, kefir, and drinking milk. This has increased the popularity of dairy products derived from small ruminants, mainly dairy sheep. The increasing popularity of these dairy products in the markets is due to the changing socio-economic status of consumers. This trend has led to an increase in the number of sheep in Türkiye (Hayaloglu and Karagul-Yuceer, 2011).

**FIGURE 2:**
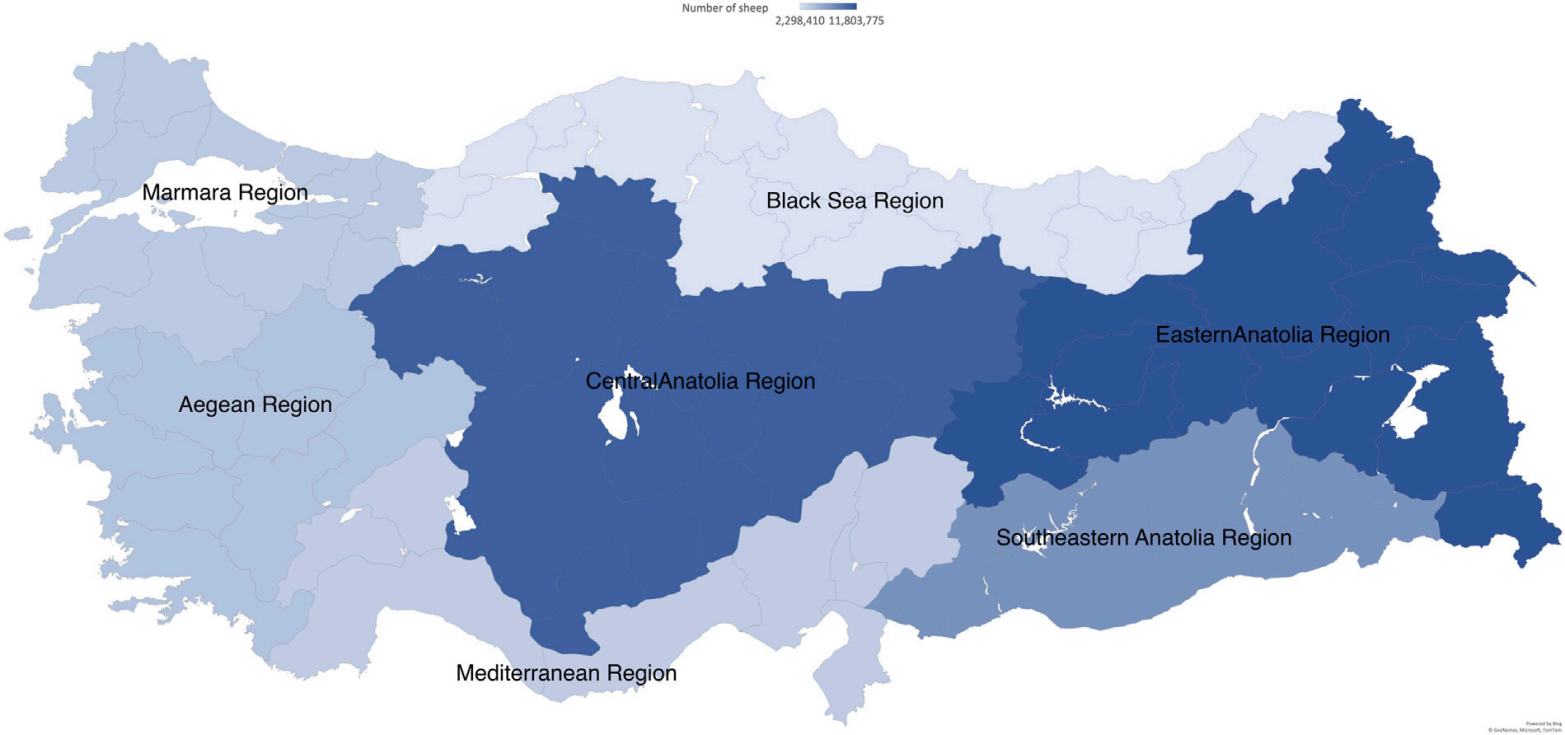
Heat map of Türkiye showing the number of sheep in seven regions in 2022. The data were obtained from the Turkish Statistical Institute.

The number of sheep in different regions of Turkey is shown in Figure 2. The 2022 data was obtained from the Turkish Statistical Institute (Turkish Statistical Institute, 2022). Türkiye is commonly divided into seven geographical regions. Out of these regions, there are five main regions where sheep are predominantly found: Central Anatolia, Eastern Anatolia, Southeastern Anatolia, Aegean, and Marmara regions. The Central Anatolia, Eastern Anatolia, and Southeastern Anatolia regions are arid and semi-arid (Ankarali, 1988). The number of sheep varies among the regions, with 10,946,183 in Central Anatolia, 11,803,775 in Eastern Anatolia, 7,608,111 in Southeastern Anatolia, 4,379,581 in Aegean, and 3,886,642 in Marmara. The Mediterranean region has the highest number of goats, possibly due to its mountainous terrain and consumer demand for goat meat.

**FIGURE 3:**
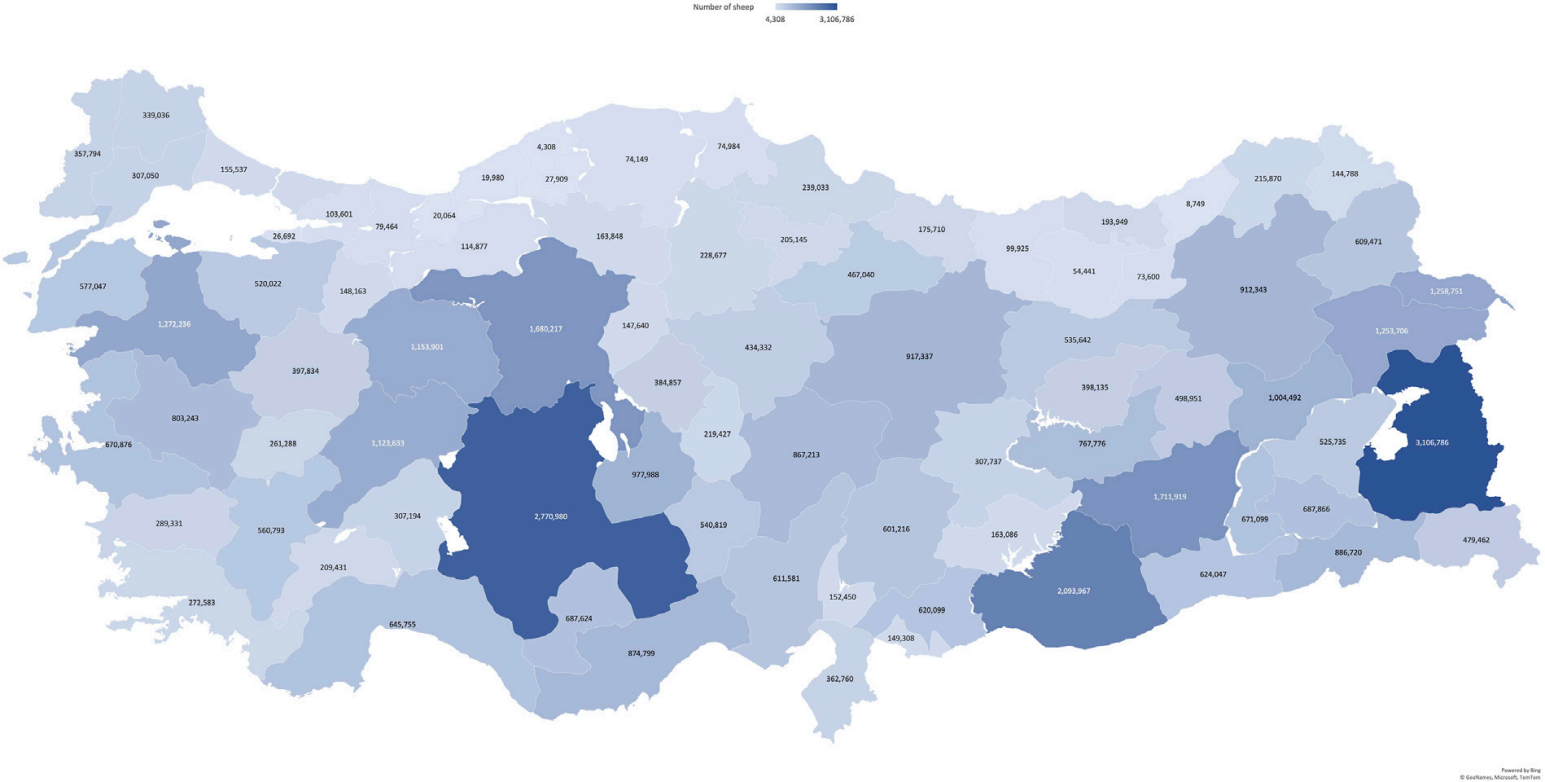
Heat map of Türkiye showing the number of sheep in each province in 2022. The data were obtained from the Ministry of Agriculture and Forestry.

The number of sheep in each province of Turkey is shown in Figure 3 using the 2022 data from the Turkish Ministry of Agriculture and Forestry. Van is the province with the highest number of sheep, totaling 3,106,786. The Eastern Anatolia region, covering the provinces of Van, Mus, Agri, Erzurum, and Bitlis, is mainly known for small-scale sheep breeding for meat and milk, with vast grazing areas consisting of meadows and pastures. Indigenous breeds known for their modest productivity are primarily used for livestock production in this region. The Central Anatolia region, encompassing the provinces of Ankara, Konya, Eskisehir, Sivas, and Karaman, is renowned for housing the highest number of sheep breeds for meat. The milk-producing Awassi breed of sheep is known to be primarily reared in the Southeastern Anatolia region, which includes Sanliurfa and Diyarbakir. Awassi sheep have become widespread around the world due to the increasing preference for sheep milk in recent years. Dairy and meat sheep breeding is well-known in the Aegean region, which includes the provinces of Izmir, Manisa, Balikesir, and Usak. The most common breed in this region is the Chios (Sakiz) breed. The Marmara region, which includes several provinces such as Kirklareli, Edirne, Bursa, Yalova, and Çanakkale, is primarily known for its substantial sheep population that is utilized for dairy, wool, and meat purposes. The common meat type breed reared in the region is the Kivircik sheep, which is known for its meat quality (Aksoy and Yavuz, 2012).

## Number of farms and farm size

**FIGURE 4:**
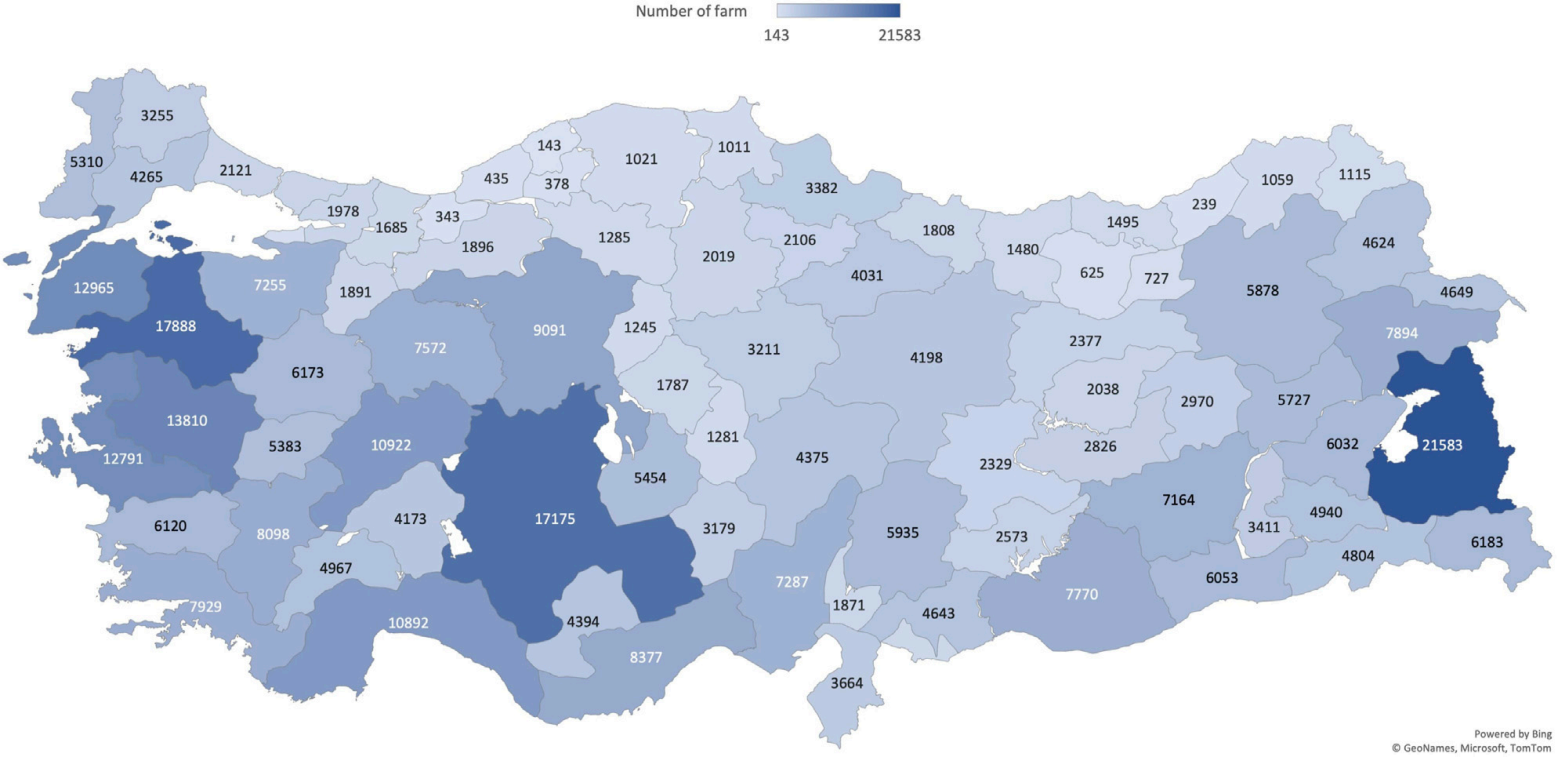
Heat map of Türkiye representing the number of farms in each province in 2022. The data were obtained from the Ministry of Agriculture and Forestry.

**FIGURE 5:**
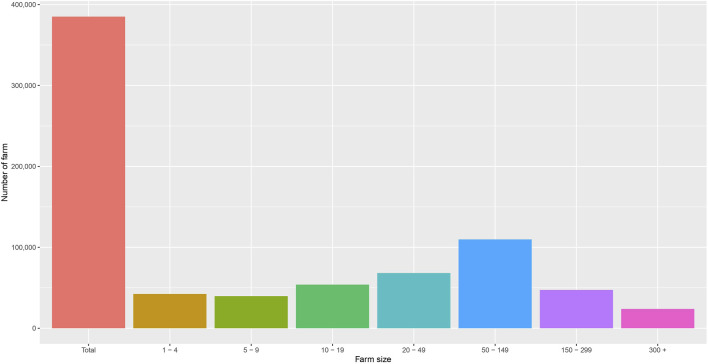
Bar graph showing the relationship between the number of farms and farm size in Türkiye in 2022. The data were obtained from the Turkish Statistical Institute.

The number of sheep farms in each province is shown in Figure 4. There are 385,071 farms in total. The provinces with the highest numbers of farms were Van (21,583), Balikesir (17,888), Konya (17,175), Manisa (13,810), and Canakkale (12,965). In Figure 5, the relationship between the number of farms and farm size in Türkiye in 2022 is shown (Turkish Statistical Institute, 2022). The majority of them are smallholder family farms. A smallholder farming system is the basic unit of sheep production in Türkiye, with family members providing most of the farm labor. The majority of farms have between 50 and 149 sheep (28.5%), with the second largest category ranging from 20 to 49 sheep (17.7%). Farms with a capacity of one to four sheep aim to meet the family’s needs for milk, butter, and cheese by rearing animals within or near the household, rather than for sale. Farms with higher capacity are managed for commercial purposes. Recently, the size of Turkish sheep flocks has been increasing, with an average flock size of 85. For comparison, the corresponding population demographics and the number and size of goat farms are shown in Supplementary Figures S1–S3.

## Sheep breeds in Türkiye

**FIGURE 6:**
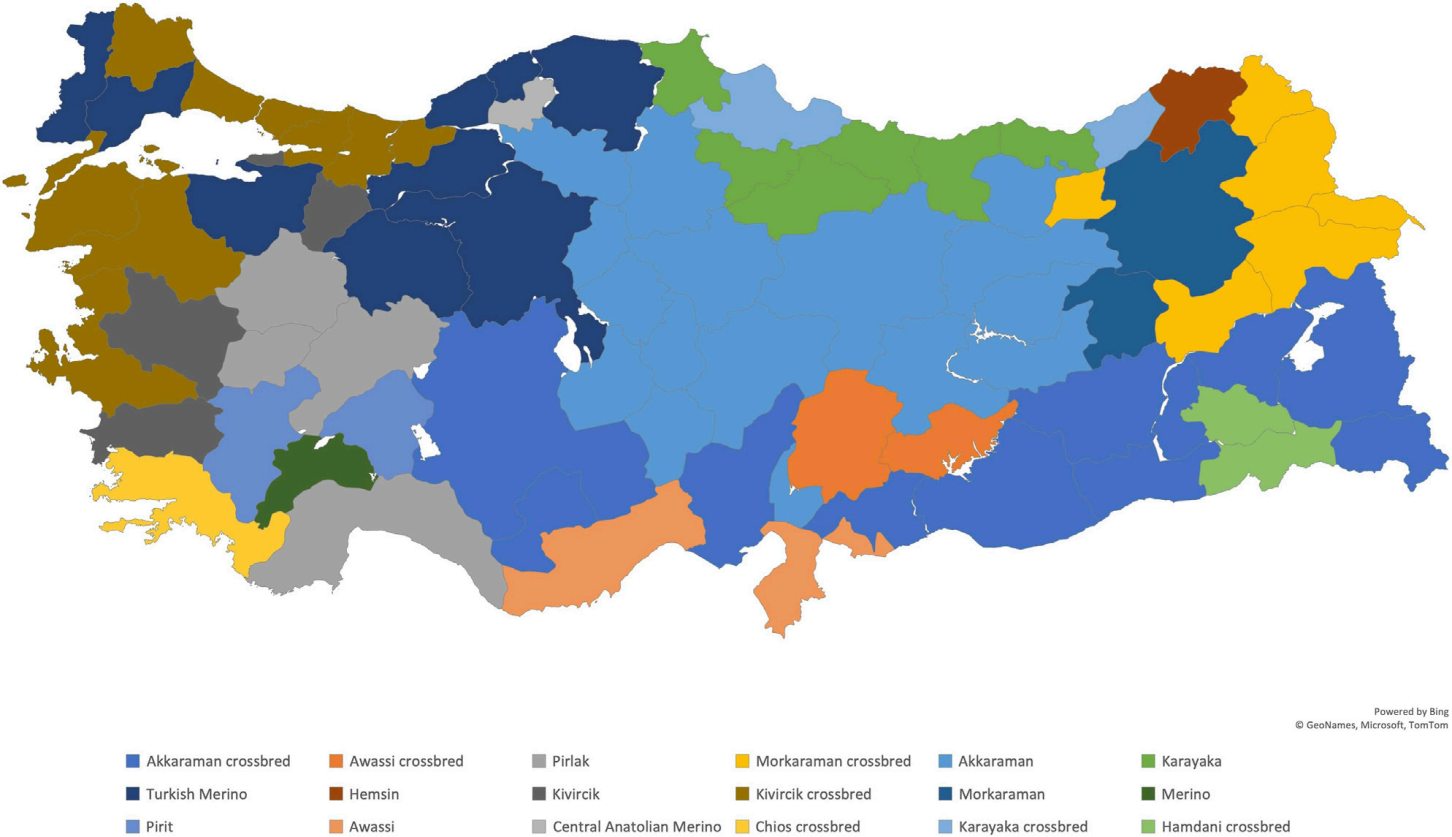
The names of the indigenous Turkish sheep breeds most represented in each province. The data were obtained from the Ministry of Agriculture and Forestry.

Türkiye is home to 33 different breeds of sheep (Sheep and Goat Breeders’ Associations of Turkey, 2021). The majority of sheep in Türkiye are considered fat-tailed, which means that they have long tails with a fat base. The tails of fat-tailed sheep in Türkiye reach their maximum size before the winter season. This enables them to survive harsh winter conditions and poor feeding periods by utlizing the fat stored in their tails (Yilmaz et al., 2012; 2013). In recent years, European sheep breeds have been increasingly bred for dairy and meat, such as Romanov, Ille de France, German Merino, Lacaune, and Assaf. However, they are primarily raised under indoor conditions. The names of the indigenous Turkish sheep breeds most represented in each province are shown in Figure 6.

### Dual-purpose breeds

Most of the sheep breeds raised for meat production are categorized as dual-purpose breeds. The two most popular breeds are the Akkaraman (White Karaman) and Morkaraman (Red Karaman) sheep, which represent approximately 65% of the total sheep population in Türkiye (Gürsoy, 2006; Uzun et al., 2006). The Akkaraman is the most commonly raised fat-tail dual-purpose breed, which is predominantly found in the Central Anatolia region. It has been reported that 45% of domestic sheep in Türkiye are Akkaraman (Ozmen et al., 2020). They are known for their adaptability to arid environments, cold resilience, disease resistance or tolerance, and ability to thrive on low-quality pastures (Arzik et al., 2022a). The Karakas in Eastern Anatolia, Kangal in Central Anatolia, Savak in Eastern Anatolia, Guney Karaman in the Mediterranean, and Norduz in the Eastern Anatolian regions are recognized as varieties/subtypes of the Akkaraman (Ozmen et al., 2020). Among them, the Guney Karaman, Norduz, and Karakas breeds are considered endangered (Yilmaz et al., 2012). The Morkaraman sheep is also a dual-purpose breed, distinguished by its fat-tailed appearance, and is primarily raised in the Eastern Anatolian region. The Daglic sheep is commonly raised in the Central Anatolia and Aegean regions. The Tuj (Tushin) sheep, with its short tail, comes from the Caucasus region and can be found throughout northeastern Türkiye. The Hemsin sheep is also a fat-tailed sheep limited to a specific area along the eastern Black Sea coast and in northeastern Türkiye (Uzun et al., 2006). Additional uncommon fat-tailed sheep breeds include the Hamdani in the Eastern and Southeastern Anatolia regions, the Hasak in the Central Anatolia region, and the Acipayam and Cine Capari (endangered) in the Aegean region.

The main indigenous thin-tailed sheep breeds in Türkiye are the Kivircik and Karayaka. The Kivircik sheep, which makes up 6%–7% of the sheep population in Türkiye, is renowned for its meat quality and has its origin in Romania and Balkan countries (Öner et al., 2014). They are mainly bred in the Marmara and Aegean regions (Öner et al., 2014). The Karayaka sheep, known for their meat quality, are dual-purpose sheep recognizable by their thin, long tails. They are relatively small in size and are primarily raised in the Black Sea region, where they have developed adaptation traits to thrive in the region’s rainy climate (Uzun et al., 2006). Other thin-tailed breeds with smaller populations include the Bafra, Bandirma, Pirlak, Karya, Gokceada, Tahirova, Hasmer, Polatli, Esme, and Sonmez in the Aegean region.

### Dairy production breeds

In general, sheep milk has a high dry matter and fat content. The Awassi and Chios breeds are known for their milk production and high fertility. The Awassi breed is characterized by a fatty tail, while the Chios breed has a thin tail, and both are raised extensively in Türkiye. These sheep are highly adaptable and excel in milk production, making them a crucial asset for many farmers with limited resources (Haile et al., 2019).

### Wool production breeds

Most of the wool-type sheep breeds in Türkiye were developed through crossbreeding. In the 1930s, the German Mutton Merino sheep were imported to Türkiye with the aim of improving the overall performance and wool quality of the local sheep breeds (Yalçın, 1986; Ankarali, 1988). The Karacabey State Farm in the South Marmara region developed the Turkish Merino (Karacabey Merino) sheep by crossing the German Mutton Merino with the Kivircik as part of a breeding program. The aim of this initiative was to improve the characteristics of the local sheep by incorporating desirable traits from the German Mutton Merino breed. The Turkish Merino is approximately 95% German Mutton Merino and 5% Kivircik (Yalçın, 1986). Although the Turkish Merino breed is known for its dual-purpose nature, it excels in wool production and mothering ability. The current population of the Turkish Merinos is approximately 4 million. It has been reported that the fleece quality of the Turkish Merino sheep is comparable to Australian Merino wool standards (Atav et al., 2023). Similarly, crossbreeding between the German Mutton Merino and the Akkaraman began in the 1950s at the Konya State Farm (Konya, Türkiye), resulted in the development of the Central Anatolian Merino (Yalçın, 1986; Ankarali, 1988). The Central Anatolian Merino is approximately 80% German Mutton Merino and 20% Akkaraman (Yalçın, 1986). The Malya state farm (Kirsehir, Türkiye) also performed crossbreeding between 35% German Mutton Merino and 65% Akkaraman, resulting in the Malya breed in the Central Anatolia region (Yilmaz et al., 2012).

## Genetic diversity

Assessing the genetic diversity of populations is essential for developing genetic conservation programs and sustainable breeding strategies. This is particularly important for Türkiye because the number of some indigenous sheep breeds is decreasing due to non-systematic crossbreeding (Ozmen et al., 2020). Most of the studies on the genetic diversity of Turkish sheep have been carried out using either mitochondrial DNA or microsatellites.

Table 1 summarizes genetic diversity studies of Turkish sheep populations using mitochondrial DNA. Sample sizes ranged from 69 to 628. Sheep can be classified into five major mitochondrial haplogroups (A–E) (Hiendleder et al., 1998; Tapio et al., 2006). It has been reported that three mitochondrial haplogroups (A, B, and C) are prevalent in indigenous Turkish sheep breeds (Pedrosa et al., 2005; Meadows et al., 2007; Demirci et al., 2013; Oner et al., 2013; Kirikci et al., 2018).

**TABLE 1:** Summary of Turkish sheep genetic diversity studies using mitochondrial DNA.

References	Sample size	Breed
Pedrosa et al. (2005)	79	Akkaraman, Hemsin, Karayak
		Morkaraman, Tuj
Meadows et al. (2007)	120	Karakas, Norduz, Morkaraman
		Cine Capari, Tuj, Chios
		Karya, Karayaka
Oner et al. (2013)	135	Daglic, Kivircik, Gokceada
		Chios, Morkaraman, Awassi
		Hemsin, Karayaka, Akkaraman
Demirci et al. (2013)	628	Karayaka, Akkaraman, Gokceada
		Daglic, Morkaraman, Kivircik
		Awassi, Herik, Karakul
		Hemsin, Cine Capari
		Chios, Norduz
Kirikci et al. (2018)	69	Karayaka

Table 2 summarizes genetic diversity studies of Turkish sheep populations based on microsatellite markers, which are the most commonly used molecular information to perform genetic diversity studies in Turkish sheep. Sample sizes ranged from 64 to 594, and the number of microsatellites used ranged from 9 to 30. The first analysis of genetic diversity using microsatellites was conducted in 2006 Gutiérrez-Gil et al. (2006); Uzun et al. (2006). High within-breed variability and low inbreeding rates were observed among the Akkaraman, Morkaraman, Tuj, Hemsin, and Karayaka breeds Gutiérrez-Gil et al. (2006). A between-breed analysis indicated a close relationship between Akkaraman, Morkaraman, and Tuj, while Hemsin and Karayaka breeds were distinct from the others (Uzun et al., 2006). The Akkaraman and Morkaraman breeds exhibited the strongest genetic relatedness. The study reported the potential admixture between Awassi, Karakas, Karayaka, Morkaraman, Norduz, and Tuj breeds, with Cine Capari and Karya being separated (Yilmaz et al., 2014). Additionally, Pirlak and Turkish Merino were found to be distinct from the Kivircik sheep (Öner et al., 2014). The Gokceada and Chios, which are raised on the coast of the Aegean region, were grouped together, while the Kivircik and Turkish Merino were found to have a distinct genetic profile with evidence of admixture (Yilmaz et al., 2015). This finding aligns with the historical development of Turkish sheep breeding, as the Turkish Merino resulted from a cross between the German Mutton Merino and Kivircik breeds. Similarly, in another study, the Turkish Merino breed exhibited a distinct cluster in comparison to the Akkaraman, Guney Karaman, and Kivircik breeds (Ameur et al., 2020). Analysis of the genetic diversity of Akkaraman subtypes revealed the presence of three distinct clusters: Kangal-Akkaraman, Karakas-Akkaraman, Norduz, and Morkaraman were grouped together, while Savak-Akkaraman and Awassi were clearly separated from all the other sheep populations (Ozmen et al., 2020).

**TABLE 2:** Summary of genetic diversity studies using microsatellites in Turkish sheep.

References	Sample size	Breed	Number of microsatellites
Gutiérrez-Gil et al. (2006)	255	Akkaraman, Hemsin	30
		Karayaka, Morkaraman, Tuj	
Uzun et al. (2006)	255	Akkaraman, Hemsin	30
		Karayaka, Morkaraman, Tuj	
Cemal et al. (2013)	123	Cine Capari	10
Yilmaz et al. (2014)	204	Awassi, Cine Capari	18
		Karakas, Karya	
		Karayaka, Morkaraman	
		Norduz, Chios, Tuj	
Öner et al. (2014)	165	Kivircik, Turkish Merino, Pirlak	15
Yilmaz et al. (2015)	250	Gokceada, Kivircik, Chios	17
		Turkish Merino	
Ozmen et al. (2020)	594	Karakas, Kangal, Savak	29
		Morkaraman, Awassi, Norduz	
Karsli et al. (2020)	120	Guney Karaman, Kangal	21
		Norduz, Karakas	
Ameur et al. (2020)	120	Akkaraman, Guney Karaman	14
		Turkish Merino, Kivircik	
Kirikci et al. (2020)	64	Karayaka	9

Subpopulation analysis of individual breeds was also performed. For example, although the Guney Karaman, Norduz, Kangal, and Karakas are considered to be varieties of the Akkaraman, clustering results showed that the Guney Karaman and Norduz are genetically different from the Kangal and Karakas. This may be due to different breeding practices and environmental conditions over the course of many years (Karsli et al., 2020). The inbreeding coefficients of the Kangal (0.26) and Guney Karaman (0.24) were higher than those of Karakas (0.16) and Norduz (0.17), which may require some management to reduce inbreeding (Karsli et al., 2020). A study of Karayaka sheep subpopulations scattered in four provinces showed that they are genetically different from each other, suggesting that the Karayaka has discrete subpopulations (Kirikci et al., 2020). In addition, the analysis of three different flocks of the Cine Capari showed high genetic variability based on unique allele numbers in different loci (Cemal et al., 2013).

## Pedigree- and genomic-based variance component analysis

Quantifying the magnitude of variance components is critical to assess the proportion of phenotypic variance controlled by genetics (i.e., heritability estimates) for each economically important trait to design effective breeding objectives.

### Growth and linear type traits

**FIGURE 7:**
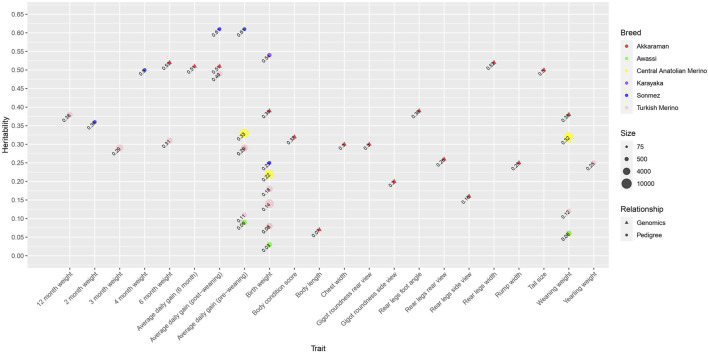
Bubble plot of pedigree- and genomic-based heritability estimates of Turkish sheep for growth and linear type traits collected from the literature. The size of the bubble is proportional to the size of the sample. Shapes and colors indicate different types of genetic relationship matrices and breeds.

Growth traits have been extensively investigated in genetic studies due to the increasing demand for sheep meat production in Türkiye. Figure 7 shows a bubble plot of pedigree and genomic heritability estimates of Turkish sheep for growth traits collected from the literature (Table 3). The heritability analyses for growth traits were conducted for six breeds, namely, the Akkaraman, Awassi, Central Anatolian Merino, Karayaka, Sonmez, and Turkish Meriono. Overall, the Akkaraman and Turkish Merino were the two most studied breeds. Most papers reported pedigree-based heritability estimates, with genome-based estimates being rare (Figure 7). The Akkaraman breed is the only one with genomic heritability estimates. A common model used in the literature included additive genetic, maternal genetic, and maternal environmental effects. Growth traits were the most studied traits for variance component analysis. For example, there were 9, 6, 5, and 3 papers reporting heritability estimates for birth weight, average daily gain (pre-weaning), weaning weight, and average daily gain (post-weaning), respectively (Table 3). For the major growth traits, heritability estimates ranged from 0.03 to 0.54 for birth weight (Ekiz et al., 2004; Ozcan et al., 2005; Koyuncu and Duru, 2009; Ozder et al., 2009; Taskin et al., 2012; Ulutas et al., 2013; Haile et al., 2019; Behrem, 2021; Kizilaslan et al., 2022), from 0.09 to 0.61 for average daily gain (pre-weaning) (Ozcan et al., 2005; Ozder et al., 2009; Taskin et al., 2012; Haile et al., 2019; Behrem, 2021; Kizilaslan et al., 2022), from 0.06 to 0.38 for weaning weight (Ekiz et al., 2004; Ozcan et al., 2005; Haile et al., 2019; Behrem, 2021; Kizilaslan et al., 2022), and from 0.49 to 0.61 for average daily gain (post-weaning) (Ozder et al., 2009; Taskin et al., 2012; Kizilaslan et al., 2022). Overall, estimates of genetic correlations between growth traits were moderate to high (Ozcan et al., 2005; Ozder et al., 2009; Haile et al., 2019; Kizilaslan et al., 2022). For example, genetic correlations between birth weight, weaning weight, yearling weight, and average daily gain (pre-weaning) were positive (Ozcan et al., 2005; Haile et al., 2019; Kizilaslan et al., 2022). In addition, birth weight showed moderate to high positive correlations with 2-month weight, 6-month weight, 12-month weight, and average daily gain (post-weaning) (Ozder et al., 2009). However, another study reported a negative genetic correlation between birth weight and weaning weight, and birth weight and average daily gain (pre-weaning) (Behrem, 2021).

**TABLE 3:** Summary of genetic parameter estimation studies for growth traits in Turkish sheep populations.

Trait	Breed	References
Birth weight	Turkish Merino	Ekiz et al. (2004); Ozcan et al. (2005)
	Turkish Merino	Koyuncu and Duru (2009)
	Turkish Merino	Ozder et al. (2009)
	Sonmez	Taskin et al. (2012)
	Karayaka	Ulutas et al. (2013)
	Awassi	Haile et al. (2019)
	Central Anatolian Merino	Behrem (2021)
	Akkaraman	Kizilaslan et al. (2022)
Average daily gain (pre-weaning)	Turkish Merino	Ozcan et al. (2005); Ozder et al. (2009)
	Sonmez	Taskin et al. (2012)
	Awassi	Haile et al. (2019)
	Central Anatolian Meri	Behrem (2021)
	Akkaraman	Kizilaslan et al. (2022)
Average daily gain (post-weaning)	Turkish Merino	Ozder et al. (2009)
	Sonmez	Ozder et al. (2009)
	Akkaraman	Kizilaslan et al. (2022)
Average daily gain (6 months)	Akkaraman	Kizilaslan et al. (2022)
Weaning weight	Turkish Merino	Ekiz et al. (2004); Ozcan et al. (2005)
	Awassi	Haile et al. (2019)
	Central Anatolian Merino	Behrem (2021)
	Akkaraman	Kizilaslan et al. (2022)
Yearling weight	Turkish Merino	Ozcan et al. (2005)
Two month weight	Sonmez	Taskin et al. (2012)
Three month weight	Turkish Merino	Ozder et al. (2009)
Four month weight	Sonmez	Taskin et al. (2012)
Six month weight	Turkish Merino	Ozder et al. (2009)
	Akkaraman	Kizilaslan et al. (2022)
Twelve month weight	Turkish Merino	Ozder et al. (2009)

**FIGURE 8:**
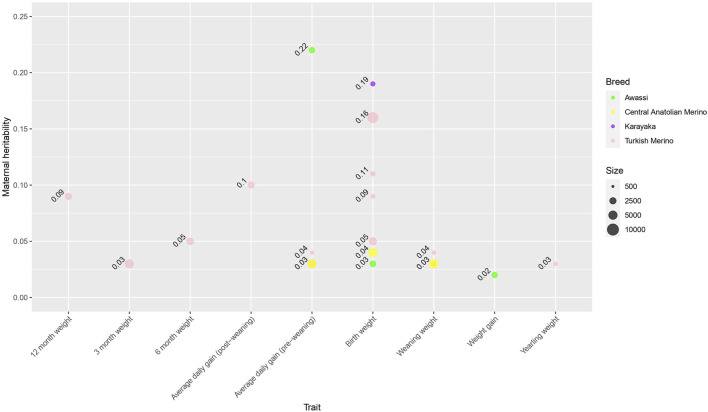
Bubble plot of pedigree-based maternal heritability estimates of Turkish sheep for growth traits collected from the literature. The size of the bubble is proportional to the sample size. Colors indicate different types of relationship breeds.

Figure 8 shows a bubble plot of maternal heritability estimates for growth traits found in the literature. The influence of maternal effects has been most studied for birth weight, with low to moderate maternal heritability estimates (Figure 8). Negative genetic correlations between additive and maternal effects have been reported for growth traits (Ekiz et al., 2004; Ozcan et al., 2005; Koyuncu and Duru, 2009; Ozder et al., 2009; Ulutas et al., 2013), which is consistent with the literature from non-Turkish sheep breeds (Maria et al., 1993; Tosh and Kemp, 1994). It has been argued that a negative genetic correlation between additive and maternal genetic effects may inhibit an increase in species size (Cundiff, 1972). Also, maternal permanent environmental variance was an important source of variation in birth weight (Ekiz et al., 2004; Ozcan et al., 2005; Koyuncu and Duru, 2009; Haile et al., 2019). To date, genomic data has not been utilized to estimate the maternal contribution to variance components.

There was only one paper that reported genomic heritability estimates for linear type traits (Figure 7; Table 4), which was performed only in the Akkaraman (Kizilaslan et al., 2022). Linear type traits showed low to moderate genomic heritability estimates ranging from 0.07 to 0.52 (Kizilaslan et al., 2022). Positive and high genetic correlations were reported between growth and linear type traits, indicating that linear type traits serve as a useful indicator for growth traits (Kizilaslan et al., 2022). No pedigree-based heritability analysis was found in the literature.

**TABLE 4:** Summary of genetic parameter estimation studies for linear type traits in Turkish sheep populations.

Trait	Breed	References
Body condition score	Akkaraman	Kizilaslan et al. (2022)
Tail size	Akkaraman	Kizilaslan et al. (2022)
Rear legs rear view	Akkaraman	Kizilaslan et al. (2022)
Gigot roundness rear view	Akkaraman	Kizilaslan et al. (2022)
Rump width	Akkaraman	Kizilaslan et al. (2022)
Rear legs width	Akkaraman	Kizilaslan et al. (2022)
Rear legs foot angle	Akkaraman	Kizilaslan et al. (2022)
Gigot roundness side view	Akkaraman	Kizilaslan et al. (2022)
Rear legs side view	Akkaraman	Kizilaslan et al. (2022)
Body length	Akkaraman	Kizilaslan et al. (2022)
Chest width	Akkaraman	Kizilaslan et al. (2022)

### Milk traits

**FIGURE 9:**
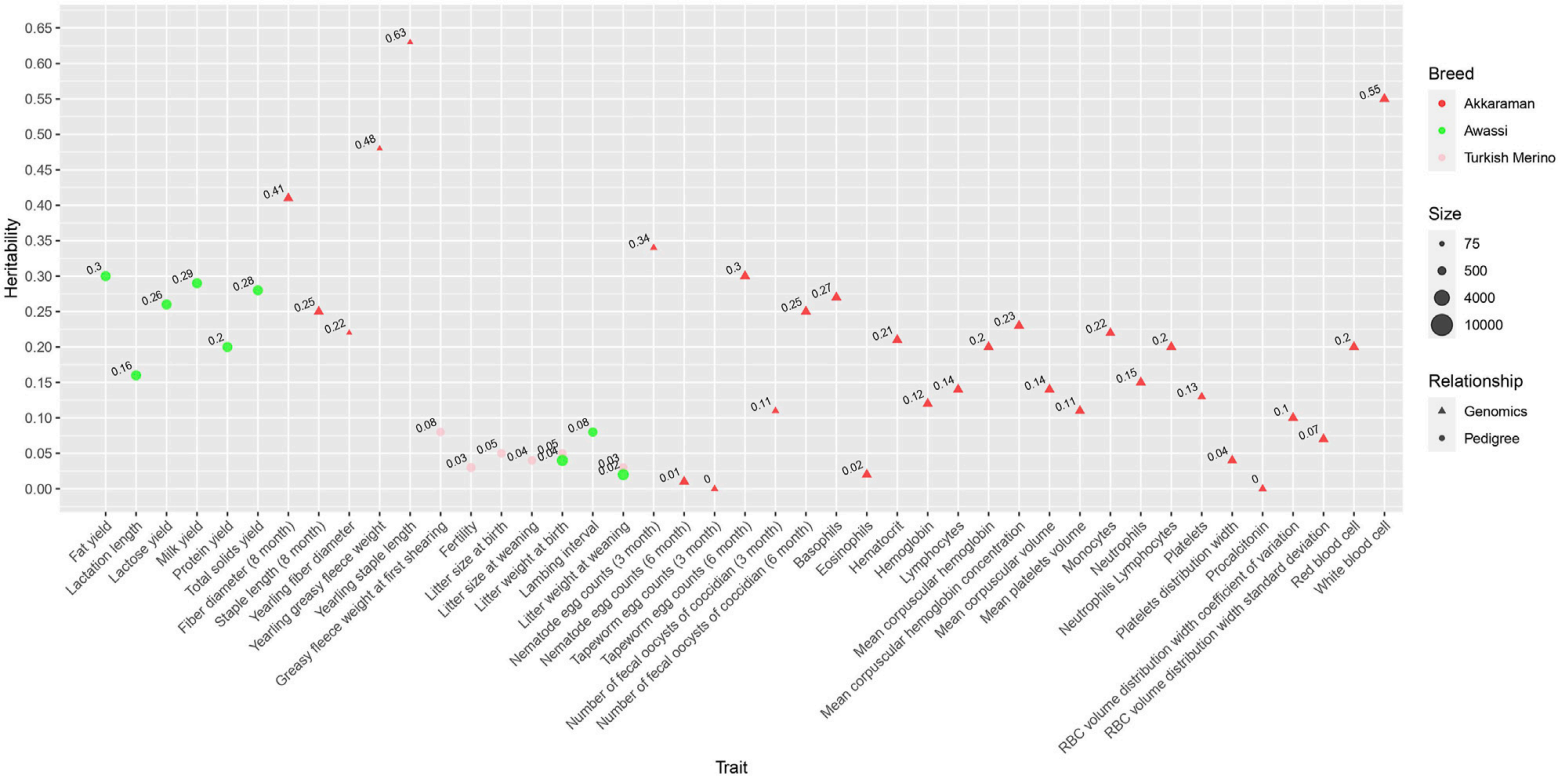
Bubble plot of heritability estimates of Turkish sheep for milk, wool, reproduction, gastrointestinal parasite resistance, and blood traits collected from the literature. The size of the bubble is proportional to the sample size. Shapes and colors indicate different types of genetic relationship matrices and breeds.

There was only one paper reporting pedigree-based heritability estimates for milk traits from the Turksih Awassi (Haile et al., 2019) (Table 5). Overall, pedigree-based heritability estimates for milk traits in the Awassi ranged from low to moderate (Figure 9). In particular, milk yield (0.29) and lactation length (0.16) had the highest and lowest heritability estimates, respectively.

**TABLE 5:** Summary of genetic parameter estimation studies for milk, wool, and reproduction traits in Turkish sheep populations.

Trait	Breed	References
Lactation length	Awassi	Haile et al. (2019)
Milk yield	Awassi	Haile et al. (2019)
Fat yield	Awassi	Haile et al. (2019)
Protein yield	Awassi	Haile et al. (2019)
Total solids yield	Awassi	Haile et al. (2019)
Lactose yield	Awassi	Haile et al. (2019)
Greasy fleece weight	Turkish Merino	Ozcan et al. (2005)
Fiber diameter (8 months)	Akkaraman	Arzik et al. (2023)
Staple length (8 months)	Akkaraman	Arzik et al. (2023)
Yearling fiber diameter	Akkaraman	Arzik et al. (2023)
Yearling staple length	Akkaraman	Arzik et al. (2023)
Yearling greasy fleece weight	Akkaraman	Arzik et al. (2023)
Fertility	Turkish Merino	Ekiz et al. (2005)
Lambing interval	Awassi	Haile et al. (2019)
Litter size at birth	Turkish Merino	Ekiz et al. (2005)
Litter weight at birth	Turkish Merino	Ekiz et al. (2005)
	Awassi	Haile et al. (2019)
Litter size at weaning	Turkish Merino	Ekiz et al. (2005)
Litter weight at weaning	Turkish Merino	Ekiz et al. (2005)
	Awassi	Haile et al. (2019)

### Wool traits

Heritability estimates for wool traits were reported in the Turkish Merino and Akkaraman (Ozcan et al., 2005; Arzik et al., 2023) (Table 5). The pedigree-based heritability estimate for greasy fleece weight was 0.08 (Figure 9). Greasy fleece weight showed a high favorable genetic correlation with birth weight, weaning weight, and average daily gain, indicating that selection for growth traits is expected to result in increased greasy fleece weight (Ozcan et al., 2005). Overall, genomic heritability estimates for wool were higher, ranging from 0.22 to 0.63 (Arzik et al., 2023).

### Reproduction traits

Heritability estimates for reproductive traits in the Turkish Merino and Awassi were all low, ranging from 0.03 to 0.08, suggesting a strong influence of environmental factors (Ekiz et al., 2005; Haile et al., 2019) (Figure 9; Table 5). Lambing interval showed a large negative genetic correlation with litter weight at birth and litter weight at weaning (Haile et al., 2019). There was also a high genetic correlation between litter weight at birth and litter weight at weaning. Although lambing interval showed moderate correlations with milk traits, litter weight at birth and litter weight at weaning exhibited negative correlations with milk traits (Haile et al., 2019).

### Gastrointestinal parasite resistance and blood traits

Heritability estimates for some less studied traits were also available in the literature (Figure 9; Table 6). Genomic heritability estimates for gastrointestinal parasite resistance, such as nematode egg count, tapeworm egg count, and coccidian fecal oocyst count, were recently reported in the Akkaraman (Arzik et al., 2022b). These estimates were low to moderate, ranging from 0 to 0.34. Similarly, genomic heritability estimates for blood traits collected to evaluate general health status in the Akkaraman ranged from low to moderate, with white blood cell showing the largest heritability of 0.55 (Arzik et al., 2022a).

**TABLE 6:** Summary of genetic parameter estimation studies for gastrointestinal parasite resistance and blood traits in Turkish sheep populations.

Trait	Breed	References
Nematode egg counts (3 months)	Akkaraman	Arzik et al. (2022b)
Nematode egg counts (6 months)	Akkaraman	Arzik et al. (2022b)
Tapeworm egg counts (3 months)	Akkaraman	Arzik et al. (2022b)
Tapeworm egg counts (6 months)	Akkaraman	Arzik et al. (2022b)
Number of fecal oocysts of coccidian (3 months)	Akkaraman	Arzik et al. (2022b)
Number of fecal oocysts of coccidian (6 months)	Akkaraman	Arzik et al. (2022b)
Basophils	Akkaraman	Arzik et al. (2022a)
Eosinophils	Akkaraman	Arzik et al. (2022a)
Hematocrit	Akkaraman	Arzik et al. (2022a)
Hemoglobin	Akkaraman	Arzik et al. (2022a)
Lymphocytes	Akkaraman	Arzik et al. (2022a)
Mean corpuscular hemoglobin	Akkaraman	Arzik et al. (2022a)
Mean corpuscular hemoglobin concentration	Akkaraman	Arzik et al. (2022a)
Mean corpuscular volume	Akkaraman	Arzik et al. (2022a)
Mean platelets volume	Akkaraman	Arzik et al. (2022a)
Monocytes	Akkaraman	Arzik et al. (2022a)
Neutrophils	Akkaraman	Arzik et al. (2022a)
Neutrophils Lymphocytes	Akkaraman	Arzik et al. (2022a)
Platelets	Akkaraman	Arzik et al. (2022a)
Platelets distribution width	Akkaraman	Arzik et al. (2022a)
Procalcitonin	Akkaraman	Arzik et al. (2022a)
RBC volume distribution width coefficient of variation	Akkaraman	Arzik et al. (2022a)
RBC volume distribution width standard deviation	Akkaraman	Arzik et al. (2022a)
Red blood cell	Akkaraman	Arzik et al. (2022a)
White blood cell	Akkaraman	Arzik et al. (2022a)

## Genome-wide association studies

**FIGURE 10:**
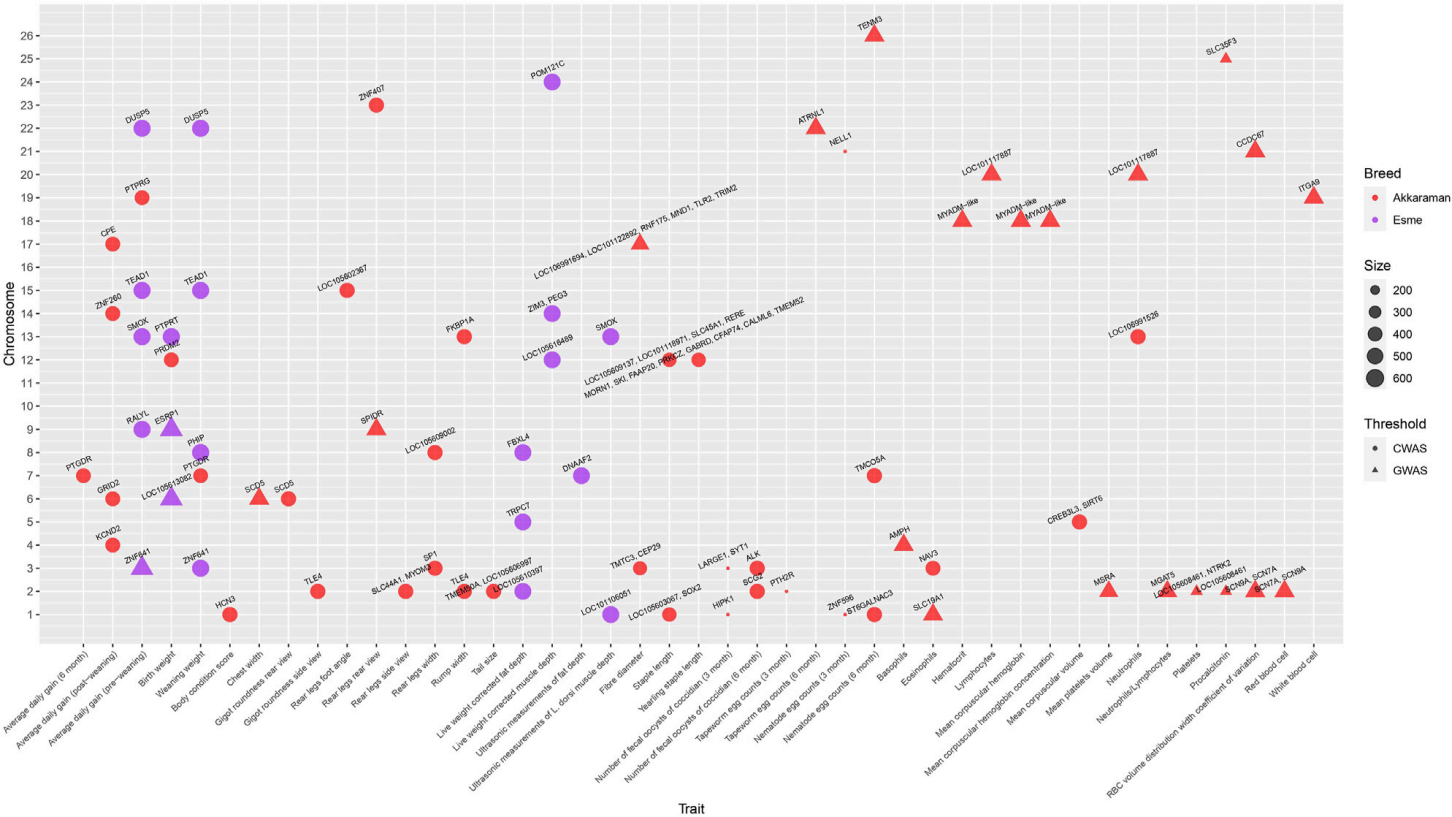
Bubble plot of important genes for growth, liner type, carcass composition, wool, gastrointestinal parasite resistance and blood traits reported in the literature. Bubble size is proportional to sample size. Colors represent CWAS: Chromosome-wide association anlaysis. GWAS: Genome-wide association analysis.

Figure 10 shows candidate genes reported in the GWAS literature for a variety of traits in Turkish sheep. There were four genome-wide association analysis papers performed on two breeds, the Akkaraman and Esme (Table 7). A total of 89 unique genes were reported for 43 traits, including growth, linear type, carcass composition, wool, gastrointestinal parasite resistance, and blood traits. Some genes were reported for more than one trait. More than half of the genes reported were significant at a chromosome-wide level rather than a genome-wide level, probably due to the small sample size used in the association analyses. Chromosome 2 had the largest number of candidate genes, followed by chromosomes 12, 3, and 1, while no genes were reported for chromosomes 10, 11, and 16.

**TABLE 7:** Summary of association analysis studies in Turkish sheep populations.

References	Breed	Genotyping (K)	Trait
Kizilaslan et al. (2022)	Akkaraman	Axiom 50	Growth and linear type
Yilmaz et al. (2022)	Esme	Illumina 50	Growth and *in vivo* carcass composition
Arzik et al. (2023)	Akkaraman	Axiom 50	Wool
Arzik et al. (2022b)	Akkaraman	Axiom 50	Gastrointestinal parasite resistance
Arzik et al. (2022a)	Akkaraman	Axiom 50	Blood

Overall, multiple genes were found for each trait in the growth and carcass composition categories. In contrast, most of the linear type, wool, gastrointestinal parasite resistance, and blood traits had one or two genes identified. Growth traits were the only group of traits studied in both breeds. Candidate genes were found for both Akkaraman and Esme for birth weight and average daily gain (pre-weaning), but there was no overlap. A number of genes were suggested for possible influence on several growth, linear type, and carcass composition traits. For example, *PTGDR* was associated with weaning weight and average daily gain (6 months), *SCD5* with chest width and gigot roundness rear view, *TLE4* with rump width and gigot roundness side view, *ZNF641*, *DUSP5*, and *TEAD1* for average daily gain (pre-weaning) and weaning weight, *SMOX* for average daily gain (pre-weaning) and ultrasonic measurements of *Longissimus dorsi* muscle depth, and *MYADM* for hematocrit, mean corpuscular volume, and mean corpuscular volume concentration. Some of these genes have also been reported in non-Turkish sheep breeds based on selection signature analysis or GWAS (Moradi et al., 2012; Xu et al., 2021).

## Discussion

In this study, we reviewed the populations, breeds, and genetic analysis of sheep populations in Türkiye. Sheep are an important small ruminant that provide income and play an important role in the livelihood of Turkish farmers. Their breeds represent a wide genetic and phenotypic diversity adapted to different environments in semi-arid and highland areas. The importance of sheep breeding is anticipated to increase due to the consistent rise in the number of sheep over the past decade.

### Past genetic studies

Genetic diversity studies are among the earliest genetic analyses conducted in Turkish sheep. While mitochondrial DNA and microsatellites have been used for genetic diversity analyses, the use of single nucleotide polymorphisms (SNPs) has not been explored. Further studies utilizing high-density SNPs distributed throughout the chromosomes are needed to obtain a more detailed evaluation of the level of genetic differentiation and to verify the findings obtained from the analyses of mitochondrial DNA and microsatellites.

Heritability estimates were reported for a variety of traits, including growth and linear type, milk, wool, reproduction, gastrointestinal parasite resistance, and blood traits. Growth and linear type traits are central to the Turkish sheep program because of their importance for production, efficiency, and profitability. Their heritability estimates have been reported for six breeds. However, studies on the rest of the traits do not represent a wide range of sheep breeds that exist in Türkiye. For example, only one, two, two, one, and one breeds have been studied for milk, wool, reproduction, gastrointestinal parasite resistance, and blood traits, respectively. Notably, the Akkaraman is the only breed for which genomic heritability estimates have been reported. Further phenotyping and genetic data collection are necessary to conduct heritability analysis for understudied breeds.

Similar to heritability studies, GWAS results were available in limited breeds and reported only in the Akkaraman and Esme. The primary limiting factor of GWAS studies was their small sample size. All studies had less than 1,000 genotyped animals, and therefore require further evaluation. The utility of whole-genome regression methods such as BayesB, BayesC*π*, or BayesR also needs to be assessed since the only model used thus far has been single-marker regression linear mixed models. While some GWAS papers have been published, there is currently no literature available on genomic prediction analysis in Turkish sheep.

### Current challenges

A community-based public animal breeding program implemented in Türkiye since 2006 has been collecting pedigree and yield data, and contributing to the genetic improvement of local breeds to some extent. The major challenge in developing breeding programs for indigenous sheep breeds in Türkiye is the lack of comprehensive pedigree data due to limited access to artificial insemination services and the widespread practice of natural mating in smallholder farming systems. This absence of pedigree data resulting from uncontrolled mating has greatly impeded the genetic improvement of Turkish sheep using conventional structured breeding programs that rely on pedigree-based best linear unbiased prediction (BLUP) (Henderson, 1975). In addition, Türkiye shares other similar bottlenecks often observed in smallholder farming systems around the world (Kosgey et al., 2006; Kosgey and Okeyo, 2007). These include lack of genotyping infrastructure and well designed contemporary groups, insufficient farmer organizations, and management of animals with unimproved genotypes. Nonetheless, the implementation of genetic evaluation using genomic information can alleviate the problem of pedigree insufficiency, and facilitate the establishment of effective breeding programs (Mrode et al., 2018). The importance of accurate pedigree recording diminishes with the availability of genomics, as genetic relatedness at the genomic level offers more accurate estimations than pedigree data. Although animal breeding and genetics aims to understand the relationship between genome and phenome, phenotyping instruments for routine use and data management are still limited. In addition, there is a need for researchers with appropriate expertise in sheep breeding, quantitative genetics, and data science (Ducrocq et al., 2018).

### Genomic selection

Genomic selection makes selection decisions on animals using genomic prediction (Meuwissen et al., 2001), and it has been widely used to perform genomic evaluations of many livestock species (Meuwissen et al., 2016; Rupp et al., 2016). Genomic prediction produces genomic estimated breeding values of animals using molecular information, such as high-density SNPs, that is available across the entire genome. The principle idea is that genome-wide SNPs tag quantitative trait loci in the genome via linkage disequilibrium, acting as markers. This allows SNPs to capture genetic variation without having to identify all causal variants in advance. Selection of animals based on genomic estimated breeding values is expected to increase genetic gain, mainly due to the reduced generation interval and more accurate estimation of genetic parameters compared to pedigree-derived estimated breeding values. When applied in the context of the BLUP framework, genomic prediction is referred to as genomic best linear unbiased prediction. In the past, genotyping large numbers of animals was prohibitively expensive. However, recent advances in biotechnology have made the cost of genotyping animals more affordable than ever before, opening up opportunities for genomic selection when coupled with advances in statistical methods (Meuwissen et al., 2001). The gains in accuracy achieved by SNPs compared to pedigree ranged from 0.05 to 0.27 (Rupp et al., 2016). Genomic selection can be particularly useful for meat production traits, such as carcass yield, carcass composition, and meat quality traits, that are measured later in life and often require animal sacrifice. In a French sheep meat breeding program, genomic selection was reported to add value over pedigree-based selection in terms of genetic gains (Shumbusho et al., 2013) and economic returns (Shumbusho et al., 2016). It may not be a suitable choice given the difficulty of accurate pedigree recording in a systematic manner in Türkiye, but a BLUP methodology to integrate pedigree and genomic information is also available (Misztal et al., 2009). Potential quantitative trait loci detected in GWAS can be used to weight SNPs to increase prediction accuracy (Santana et al., 2023). Türkiye has started investing in molecular technologies for sheep breeding over the last 5 years. However, the application of these technologies has not yet been fully realized. Although the application of genomic selection in Turkish sheep is still in its infancy, there is no doubt that it will achieve significant success in the coming years as routinely recorded genotyping and phenotyping systems are established. In addition to production traits, future genomic selection studies should include phenotypes that are difficult or expensive to measure, such as reproductive performance, meat quality, feed efficiency, longevity, and disease susceptibility traits.

### Next steps forward

One of the first steps in implementing genomic selection is to identify the breeding objective traits and carefully define the selection criteria for the particular environments being considered. Then, sustainable and routinely recorded reliable phenotyping systems must be established, followed by a cost-benefit analysis to determine which genotyping platform to use. Currently, low, medium, and high density genotyping chips for sheep are available on the market (Rupp et al., 2016). If only selected animals can be genotyped with higher density chips, imputation can be used to impute low density genotypes to medium or high density genotypes. In addition, genotyping-by-sequencing and low-pass sequencing have been used in recent years to obtain genomic information in sheep instead of SNP chips (Dodds et al., 2021).

The critical step is to create a reference population consisting of individuals with phenotypic records and genotypes. A reference population is used to estimate marker-phenotype association, which allows prediction of genomic estimated breeding values. The development of large reference populations is necessary because accuracy and expected genetic gain are proportional to the size of the reference population. The size of purebred sheep reference populations in other countries is reported to be in the range of 2,000 to 6,000 (Rupp et al., 2016). If a reference population size for each purebred is not sufficient, multi-breed or crossbred genomic evaluations can serve as an alternative solution (Daetwyler et al., 2012). This is particularly important for Turkish sheep because crossbreeding is not uncommon to combine adaptation and production ability under various environments. In the case of Turkish sheep breeds that have been exported to other regions of the world where there is structured breeding programs, there might be opportunities for across-country genomic predictions as evaluated in New Zealand and Norwegian sheep breeds with similar development history (Oliveira et al., 2020; 2022). Sufficient DNA extraction laboratories, computing facilities, and data storage infrastructure are needed to analyze thousands of animals using whole-genome regression. In addition, the use of information and sensing technologies for phenotyping has the potential to accelerate the data collection process (Morota et al., 2018). For example, digital tools such as mobile phones and tablets have been successfully used in low- and middle-income countries to collect phenotypic data and return management information to farmers to help them make informed decisions, given the availability of reliable internet connectivity (Mrode et al., 2020). A reference population needs to be updated occasionally and genetic progress needs to be closely monitored. Collectively, a variety of phenotypic data coupled with genomic resources could be used to design comprehensive breeding objectives for sheep by developing appropriate selection indexes tailored to the needs in Türkiye. The use of genomics is also proving useful in parentage verification, determining breed composition, and better managing inbreeding because genomic data provide more accurate estimates of genetic relationships between individuals than pedigree records. Increased rates of inbreeding discovered through certain studies call for caution. For instance, the high level of inbreeding observed among Karakas and Norduz sheep requires attention.

Finally, establishing effective multi-organizational or multi-national partnerships involving all sectors of the sheep breeding chain is a key to success (Burrow et al., 2021). For example, collaboration with established resource populations in other countries with similar management systems or environmental conditions may increase the accuracy of genomic estimated breeding values or the co-development of phenotyping platforms. These resource populations and infrastructure are expected to provide opportunities for genetic improvement of flocks and bring the economic, social, and environmental benefits of genomic selection to smallholder farmers. One bottom-up approach we can learn from when introducing or transferring a new breeding program to smallholder farms is community-based breeding programs (Mueller et al., 2015). Farmers are the key players in community-based breeding programs, as opposed to centralized or government-controlled breeding programs. The feasibility of community-based breeding programs resulting in measurable genetic gains in growth traits has been reported for sheep in Ethiopia (Mirkena et al., 2012; Gizaw et al., 2014; Haile et al., 2020). An important factor to consider is the participation of farmers in the process of planning and implementation, with shared breeding objectives (Wurzinger et al., 2011). An example of farmer participation is involvement in decisions about research agendas and resource allocation. Many attempts to establish new genetic improvement programs for low-input smallholder livestock systems have failed when financial support ended or government priorities changed, if farmers were not involved in the programs or were limited to providing information to researchers. For this reason, genomic selection breeding programs should be self-sustaining in the long term.

### Conclusions

The role of sheep breeding and genetics in Türkiye in supporting the incomes and livelihoods of smallholder farmers is expected to grow as the number of sheep has increased in recent times. Historically, sheep in Türkiye were raised mainly for meat production, but there has recently been a surge in demand for dairy products with high nutritional and health benefits. Although there are some genetic studies in indigenous Turkish sheep in the literature, genetic diversity studies need to be revisited using genome-wide SNPs, and the scope of genetic parameter estimation and GWAS studies should be expanded to cover traits and breeds that have previously been overlooked. Genomic selection has not yet been applied, but it has the potential to overcome the difficulties of implementing traditional pedigree-based breeding programs where pedigree recording is required. The use of genomics also contributes to parentage verification, determination of breed composition, and better management of inbreeding. The establishment of sustainable systems for genotyping and phenotyping is vital. Given their superior adaptation to local environments, utilizing indigenous sheep breeds would improve food security and reduce environmental impact in Türkiye.
