# Supplementary material for: Review of sheep breeding and genetic research in Türkiye
Source: Front Genet. 2024 Jan 25;15:1308113. doi: 10.3389/fgene.2024.1308113 (PMC10850221; doi:10.3389/fgene.2024.1308113)
Supplement: Supplementary file 1 [file DataSheet1.pdf]

Supplementary Material

Figures

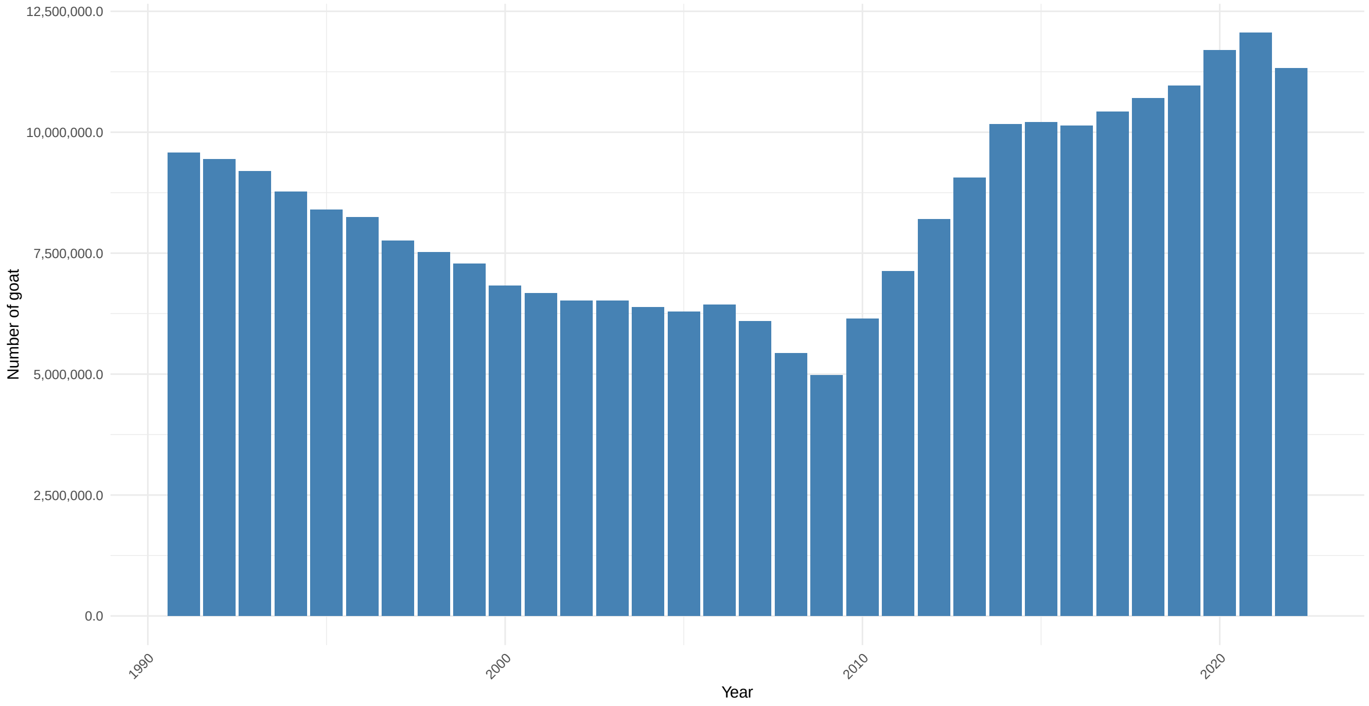

Figure S1: Bar graph showing the total number of goats in the last 30 years in Türkiye. The 2022 data were obtained from the Turkish Statistical Institute.

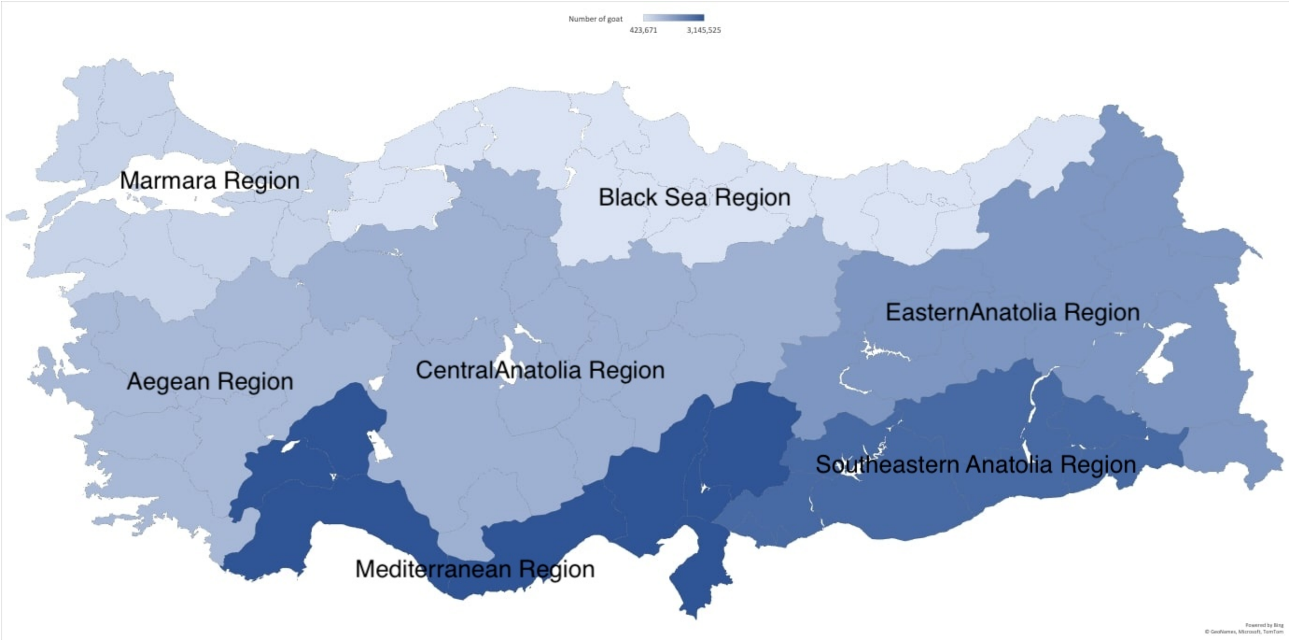

Figure S2: Heat map of Türkiye showing the number of goats in seven regions in 2022. The data were obtained from the Turkish Statistical Institute.

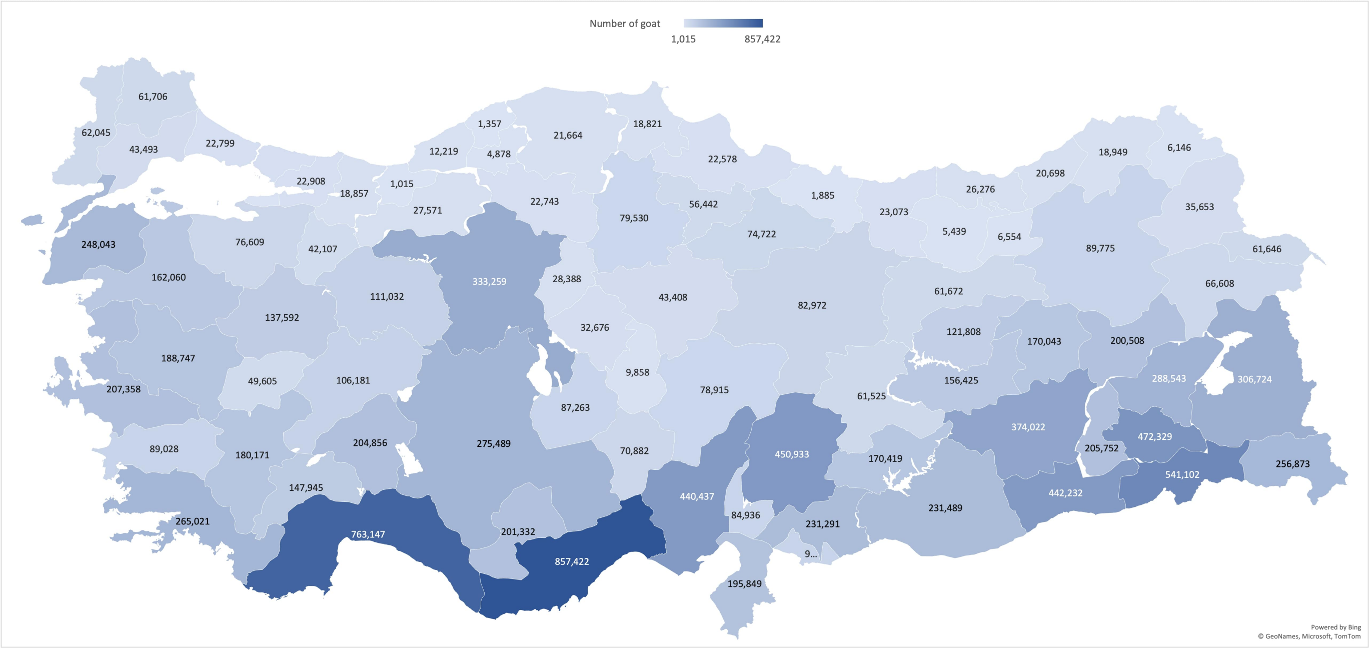

Figure S3: Heat map of Türkiye showing the number of goats in each province in 2022. The data were obtained from the the Ministry of Agriculture and Forestry.
